# Proteomics of *Aggregatibacter actinomycetemcomitans* Outer Membrane Vesicles

**DOI:** 10.1371/journal.pone.0138591

**Published:** 2015-09-18

**Authors:** Thomas Kieselbach, Vincent Zijnge, Elisabeth Granström, Jan Oscarsson

**Affiliations:** 1 Department of Chemistry, Umeå University, Umeå, Sweden; 2 Center for Dentistry and Oral Hygiene, University Medical Center Groningen, Groningen, The Netherlands; 3 Oral Microbiology, Department of Odontology, Umeå University, Umeå, Sweden; University of Oklahoma Health Sciences Center, UNITED STATES

## Abstract

*Aggregatibacter actinomycetemcomitans* is an oral and systemic pathogen associated with aggressive forms of periodontitis and with endocarditis. Outer membrane vesicles (OMVs) released by this species have been demonstrated to deliver effector proteins such as cytolethal distending toxin (CDT) and leukotoxin (LtxA) into human host cells and to act as triggers of innate immunity upon carriage of NOD1- and NOD2-active pathogen-associated molecular patterns (PAMPs). To improve our understanding of the pathogenicity-associated functions that *A*. *actinomycetemcomitans* exports via OMVs, we studied the proteome of density gradient-purified OMVs from a rough-colony type clinical isolate, strain 173 (serotype e) using liquid chromatography-tandem mass spectrometry (LC-MS/MS). This analysis yielded the identification of 151 proteins, which were found in at least three out of four independent experiments. Data are available via ProteomeXchange with identifier PXD002509. Through this study, we not only confirmed the vesicle-associated release of LtxA, and the presence of proteins, which are known to act as immunoreactive antigens in the human host, but we also identified numerous additional putative virulence-related proteins in the *A*. *actinomycetemcomitans* OMV proteome. The known and putative functions of these proteins include immune evasion, drug targeting, and iron/nutrient acquisition. In summary, our findings are consistent with an OMV-associated proteome that exhibits several offensive and defensive functions, and they provide a comprehensive basis to further disclose roles of *A*. *actinomycetemcomitans* OMVs in periodontal and systemic disease.

## Introduction

Periodontal diseases are characterized by chronic inflammation of the gingiva, and progressive destruction of alveolar bone and supporting tissues around the teeth resulting in tooth loss [1]. Colonization by the Gram-negative human pathogen *Aggregatibacter actinomycetemcomitans* is strongly associated with aggressive forms of periodontitis in adolescents and young adults [2, 3], and the organism also is a systemic pathogen, associated with non-oral infections such as endocarditis [4].

The prevalence of *A*. *actinomycetemcomitans* varies widely with geographic origin, age and life style of a population [3, 5]. Seven serotypes (a-g) exist, which form genetically divergent lineages [3, 6]. Whole genome sequencing of 14 *A*. *actinomycetemcomitans* strains has disclosed a pangenome of 3301 genes (2034 core and 1267 flexible genes), and it showed that the difference between any two strains is 0.4–19.5% of the genomic content [7]. The mechanisms by which *A*. *actinomycetemcomitans* causes periodontal attachment loss and systemic disease are not entirely known. As a highly leukotoxic clone (JP2; serotype b) is strongly linked to disease progression in North African adolescents [2, 8], leukotoxin (LtxA) may have a major role in aggressive forms of periodontitis. Like HlyA of *Escherichia coli*, LtxA is an RTX toxin, which selectively affects human cells of hematopoetic origin. It binds to the lymphocyte function associated receptor 1 (LFA-1) and causes disruption of the membrane integrity [9–12]. Moreover, similar to several other Gram-negative bacteria (e.g. *Campylobacter jejuni*, *Escherichia coli*, *Salmonella enterica*, and *Shigella dysenteriae*), *A*. *actinomycetemcomitans* produces a cytolethal distending toxin (CDT), which kills host cells including gingival fibroblasts by blocking their proliferation [13–16]. In addition to LtxA and CDT, accumulating evidence strongly suggests the importance of additional, yet undisclosed *A*. *actinomycetemcomitans* virulence mechanisms in periodontitis [3, 17, 18].

It has been evident for decades that bacteria, archaea, and eukaryotes produce membrane vesicles (MVs). Membrane vesicles (“Type Zero secretion”) represent a very basic but relevant mode of protein export by bacteria, and are released by both commensals and pathogens *in vivo* and during infection of host cells *in vitro* [19–23]. Vesicles from both Gram-negative and Gram-positive bacteria can carry out a number of offensive functions, including targeting concentrated virulence factors, and inflammatory stimulants such as LPS and peptidoglycan fragments to host cells and tissues to manipulate the host immune response [24–30]. For consistency, in this report vesicles liberated by Gram-negative organisms are referred to as outer membrane vesicles (OMVs). Biogenesis of OMVs is not known in great detail. They are generated as a result of the budding out of small portions of the outer membrane and the encapsulation of periplasmic components [31–33]. In chronic localized infections, such as periodontitis OMVs may represent an important source of inflammatory stimulants both locally and systemically, upon entry into the circulation [34, 35]. For instance, *A*. *actinomycetemcomitans* OMVs can deliver biologically active virulence factors (CDT, OmpA) into HeLa cells and human gingival fibroblasts (HGF) [36]. In addition, the export of LtxA, peptidoglycan-associated lipoprotein (Pal), and the chaperonin GroEL also involves OMVs [37–40]. We recently demonstrated that *A*. *actinomycetemcomitans* OMVs carrying NOD1- and NOD2-active peptidoglycan are internalized into non-phagocytic human cells including gingival fibroblasts [41], revealing a role of the vesicles as a trigger of innate immunity. Membrane vesicles also exhibit several defensive functions. For example, it was recently demonstrated that *Vibrio cholerae* OMVs contribute to antimicrobial peptide resistance [42], and that biologically active β-lactamase is released via vesicles in *Staphylococcus aureus* [43]. There is also evidence that *Moraxella catarrhalis* OMVs mediate immune evasion by inactivating complement factor C3 [44, 45].

Accumulating knowledge from genomic, proteomic and transcriptomic analyses of *A*. *actinomycetemcomitans* strains provides novel, comprehensive information on virulence-related properties of this organism, and represents a good molecular basis for further disclosing its pathogenicity mechanisms and role in periodontal and systemic disease [7, 18, 46–48]. In recent years, several high-throughput proteomics studies have revealed the identity of vesicle proteins in an array of bacterial species [28, 49]. However, the detailed composition of the *A*. *actinomycetemcomitans* OMV proteome was not known. To improve our understanding of the virulence potential of *A*. *actinomycetemcomitans* OMVs, we have used liquid chromatography-tandem mass spectrometry (LC-MS/MS) to identify the OMV-associated proteome of strain 173 (serotype e). This strain was selected because it was earlier assessed with respect to both production of LtxA and CDT [17, 50], which provides a basis for the analysis of unknown virulence-related proteins. In this study we identified 151 proteins in the *A*. *actinomycetemcomitans* OMV-associated proteome. In addition to confirming their immunoreactive potential, and leukotoxic activity, we provide a comprehensive overview of additional putative offensive and defensive mechanisms of the vesicles, which can serve as the groundwork for further disclosing their role in periodontal and systemic disease.

## Materials and Methods

### Bacterial strains and growth conditions

The *A*. *actinomycetemcomitans* serotype e strain, 173 (rough colony type) belongs to a strain collection sampled from an adolescent West African population, and was isolated from an individual with tooth attachment loss at baseline [50]. Strain D7SS is a smooth-colony derivative of D7S (serotype a), which was originally isolated from a patient with aggressive periodontal disease [51]. Mutant derivatives of D7SS, D7SSΔ*ltxA* Δ*cdtABC* [52], and D7SS-p (*pal*-deficient) [53] were constructed earlier. JP2 (serotype b) is a highly leukotoxic strain (serotype b) [54]. The *A*. *actinomycetemcomitans* strains were routinely cultivated in air supplemented with 5% CO_2_, at 37°C as previously described [39], on blood agar plates (5% defibrinated horse blood, 5 mg hemin/l, 10 mg Vitamin K/l, Columbia agar base).

### SDS-PAGE and Western immunoblotting

The procedures used for SDS-PAGE and immunoblot analysis have been described previously [53, 55]. Gels were stained using non-ammonical Silver-staining (BioRad). For Western blot experiments, we used a patient serum from a periodontitis subject, which exhibits strong reactivity to *A*. *actinomycetemcomitans* antigens [56] (final dilution 1:2,000). As a control, a serum sampled from a periodontally healthy, *A*. *actinomycetemcomitans*-negative individual was used (1:2,000). Polyclonal antisera made in rabbit against *E*. *coli* GroEL protein (Sigma-Aldrich), and against whole *A*. *actinomycetemcomitans* serotype e bacterial cells [57] were used at 1:8,000 and 1:10,000 final dilutions, respectively. As secondary antibody, anti-human or anti-rabbit horseradish peroxidase (HRP)-conjugate (Jackson ImmunoResearch, Newmarket, UK) was used (1:10,000). Immunoreactive bands were visualized using Clarity™ Western ECL Substrate (Bio-Rad) and the ChemiDoc™ XRS+ System (Bio-Rad).

### Isolation and purification of outer membrane vesicles

OMVs were isolated from *A*. *actinomycetemcomitans* cells harvested from an average of ten blood agar plates, using ultracentrifugation as described earlier [36, 41]. OMV pellets were washed twice with PBS (85,000 × *g*; 2 h, 4°C) using a 70 Ti rotor (Beckman Instruments Inc.), and then used as the OMV preparation. The yield of OMVs was estimated by quantifying vesicle preparations for protein content using a Picodrop™ (Picodrop Ltd.) [41]. To assess the uniformity of OMV preparations, samples were validated by atomic force microscopy (AFM), and SDS-PAGE. OMVs were also checked for absence of bacterial contamination by cultivating small aliquots on blood agar plates in air supplemented with 5% CO_2_, at 37°C for 3 days. To separate the vesicles from free or loosely associated proteins, OMV preparations were purified using density gradient centrifugation [36, 41]. In this gradient, OMVs migrate to positions equal to their density. Only proteins integral, internal, or tightly associated with membrane lipids will move significantly through the gradient [58]. For this procedure, OMV pellets were resuspended in 50 mM HEPES (pH 6.8) and then adjusted to 45% OptiPrep (Sigma-Aldrich) in a final volume of 150 μl. The sample was transferred to the bottom of a 4-ml ultracentrifuge tube, and then different OptiPrep-HEPES layers were sequentially added as follows: 900 μl of 35%, 900 μl of 30%, 660 μl of 25%, 660 μl of 20%, 400 μl of 15%, and 500 μl of 10%. Gradients were centrifuged at 180,000 × *g* (3 h, 4°C) in an SW 60 Ti rotor (Beckman Instruments Inc.), and fractions of equal volumes (200 μl) were removed sequentially from the top.

### Preparation of OMV Samples for Liquid Chromatography Mass Spectrometry (LC-MS/MS)

After SDS-PAGE analysis, selected fractions from the Optiprep density gradient were pooled into a total volume of 780 μl. Subsequently, 400 μl HEPES buffer (50 mM, pH 7.8) was added to increase the pH to >7. For reduction of disulfide bonds, dithiotreitol (DTT) was added at 50 mM final concentration, and the sample was heated for 20 min at 60°C. For the alkylation reaction, iodacetamide (IAM) was added to the sample at a final concentration of 20 mM. After 60 min treatment in the dark, the OMV proteins were precipitated using trichloroacetic acid (TCA), and stored in -20°C overnight. The next day, the sample was centrifuged in a Beckman Coulter Avanti J-20 XP centrifuge (JA 18–1 Beckman Instruments Inc., California, USA) at 16,000 × *g* for 30 min at 4°C, followed by a subsequent washing step where the sample was centrifuged at 16,000 × *g* for 15 min with 80% ethanol. Finally, the OMV pellet was dried in air, and used for the preparation of an in-solution digest for LC-MS/MS analysis.

### Preparation of in-solution digests of OMV proteins

A dried pellet of TCA-precipitated OMVs was resuspended in 15 μl fresh 8 M urea and 20 μl of 50 mM ammonium bicarbonate containing 0.2% ProteaseMax^TM^ surfactant (Promega Biotech, Nacka, Sweden). The vesicle proteins were dissolved by shaking at 150 rpm for 20 min at 37°C. After the solubilization, 50 μl of 50 mM ammonium bicarbonate, 10.4 μl of Milli Q water, 1 μl of 50 mM ammonium bicarbonate containing 1% ProteaseMax surfactant, and 3.6 μl of 0.5 μg/μl trypsin stock solution (sequencing grade trypsin, Promega Biotech, Nacka, Sweden) were added. The final concentrations were 40 mM ammonium bicarbonate, 0.05% ProteaseMax surfactant, 1.2 M urea and 18 ng/ml of trypsin in a volume of 0.1 ml. To generate peptides for mass spectrometry the in-solution digestion was performed for either 1 to 1.5 hours at 50°C or for 2 to 3 hours at 37°C [59]. Finally, the digestion was stopped by addition of 10% trifluoroacetic acid to a final concentration of 0.5–1.0%, and the peptides were desalted using homemade reversed phase micro columns [60, 61]. The bound peptides were eluted using 0.1% formic acid containing 50% acetonitrile. The solvent was removed using a speedvac and the peptides were dissolved in 0.1% formic acid for further analysis by mass spectrometry.

### LC-MS/MS analysis and data processing

The peptides generated by in-solution digestion were analyzed by LC-MS/MS (DDA, 5 MS/MS channels) using a Synapt G2 mass spectrometer (Waters, Sollentuna, Sweden) linked to a nano UPLC (Waters, Sollentuna, Sweden). Separation of the peptides was performed by C_18_ nano reversed phase chromatography (Acquity nano UPLC column 1.8 mm HSS T3 75 mm × 200 mm). The peptides were separated at a flow rate of 300 nl/min using a 4 h long linear gradient (1 to 30 percent acetonitrile for 3 h, followed by 30 to 50 percent acetonitrile for 1 h). Spectra processing was performed using the ProteinLynx Global Server 2.5.2 software (Waters, Sollentuna, Sweden) using lockspray calibration and fast de-isotoping for the MS and MS/MS mode. In addition, the spectra were also processed using the Mascot Distiller (version 2.4.3.3, Matrix Science, London, UK) and the standard settings for DDA data from Waters instruments. Database searches using the peaklist files of the processed mass spectra were performed using the Mascot search engine (version 2.4, MatrixScience, London, UK) in the database of *A*. *actinomycetemcomitans* serotype e strain SC1083, which is available at Ensembl Bacteria at: http://bacteria.ensembl.org/aggregatibacter_actinomycetemcomitans_serotype_e_str_sc1083/Info/Index. This database was selected because the genome of strain 173 was not available, and it contained the gene models from a strain of the same serotype. The search parameters permitted mass errors of 20 ppm (MS mode) and 0.1 Da (MS/MS mode), respectively. Modifications included variable oxidation of methionine, N-terminal acetylation, deamidation (N,Q) and fixed cysteine derivation by carbamidomethylation. The false discovery rate was <1%. Compilation of non-redundant protein lists was performed using the Protein Extractor of the ProteinScape server (version 3, Bruker Daltonik GmbH, Bremen, Germany). Ion scores of individual MS/MS spectra lower than 30 and Mascot protein scores lower than 100 were not considered for the compilation of the results. The LC-MS/MS analysis was performed with four independent OMV preparations. The mass spectrometry proteomics data have been deposited to the ProteomeXchange Consortium [62] via the PRIDE partner repository with the dataset identifier PXD002509 and 10.6019/PXD002509.

### Bioinformatics analysis of the LC-MS/MS results

The final result list was assembled using the proteins that were detected in at least three of the four OMV preparations that were analyzed. It included 151 proteins that were sorted according to their Clusters of Orthologous groups (COG) categories. COG groups were created manually by using the gene identifiers for searches in the COG database at http://www.ncbi.nlm.nih.gov/Structure/cdd/wrpsb.cgi. The COG classifiers were grouped according to the definitions in the conserved domains database at National Center for Biotechnology Information (NCBI) [63]. The subcellular locations of the identified proteins were predicted using the program PSORT3b 3.0 [64], and members of KEGG pathways were identified using the KOBAS 2.0 server [65] and the annotations of the *A*. *actinomycetemcomitans* strain D7S genome as a template. The proteins were also subject to *in silico* analysis using VirulentPred (http://203.92.44.117/virulent/submit.html), which predicts bacterial virulence proteins based on their sequences information [66].

### Atomic Force Microscopy

For AFM, samples of OMVs were diluted with ultrapure water (Millipore) and placed onto a freshly cleaved mica surface. Samples were incubated for 5 min at room temperature, washed with ultrapure water, and then placed in a desiccator for ~2 h in order to dry. The samples were finally magnified through a Nanoscope V Atomic Force Microscope (Bruker AXS GmbH, Karlsruhe, Germany), using tapping mode. Final images were plane fitted in both the *x* and *y* axes and are presented in amplitude mode.

### Cell cultures and determination of leukotoxic activity of OMVs

Human myeloid monocytic THP-1 (ATCC 16) cells were cultivated in RPMI 1640 (Sigma-Aldrich, St Louis, MI, USA) with 10% fetal bovine serum (FBS) (Sigma-Aldrich) at 37°C in 5% CO_2_. Leukotoxic activity of *A*. *actinomycetemcomitans* OMVs was determined by quantitating the release of lactate dehydrogenase (LDH) from treated THP-1 cells as described earlier [17, 67]. In brief, aliquots of 100μl (1×10^6^ cells per ml) of phorbol 12-myristate 13 acetate (PMA)-differentiated THP-1 cells were incubated with OMVs for 120 min such that the final OMV protein concentration was 100 μg/ml. As control treatment, PBS buffer was used. The release of LDH was expressed as % of the maximal release (100%) caused by incubation with 0.1% Triton X-100.

## Results and Discussion

### Purification of OMVs from the *A*. *actinomycetemcomitans* serotype e strain 173

**Fig 1:**
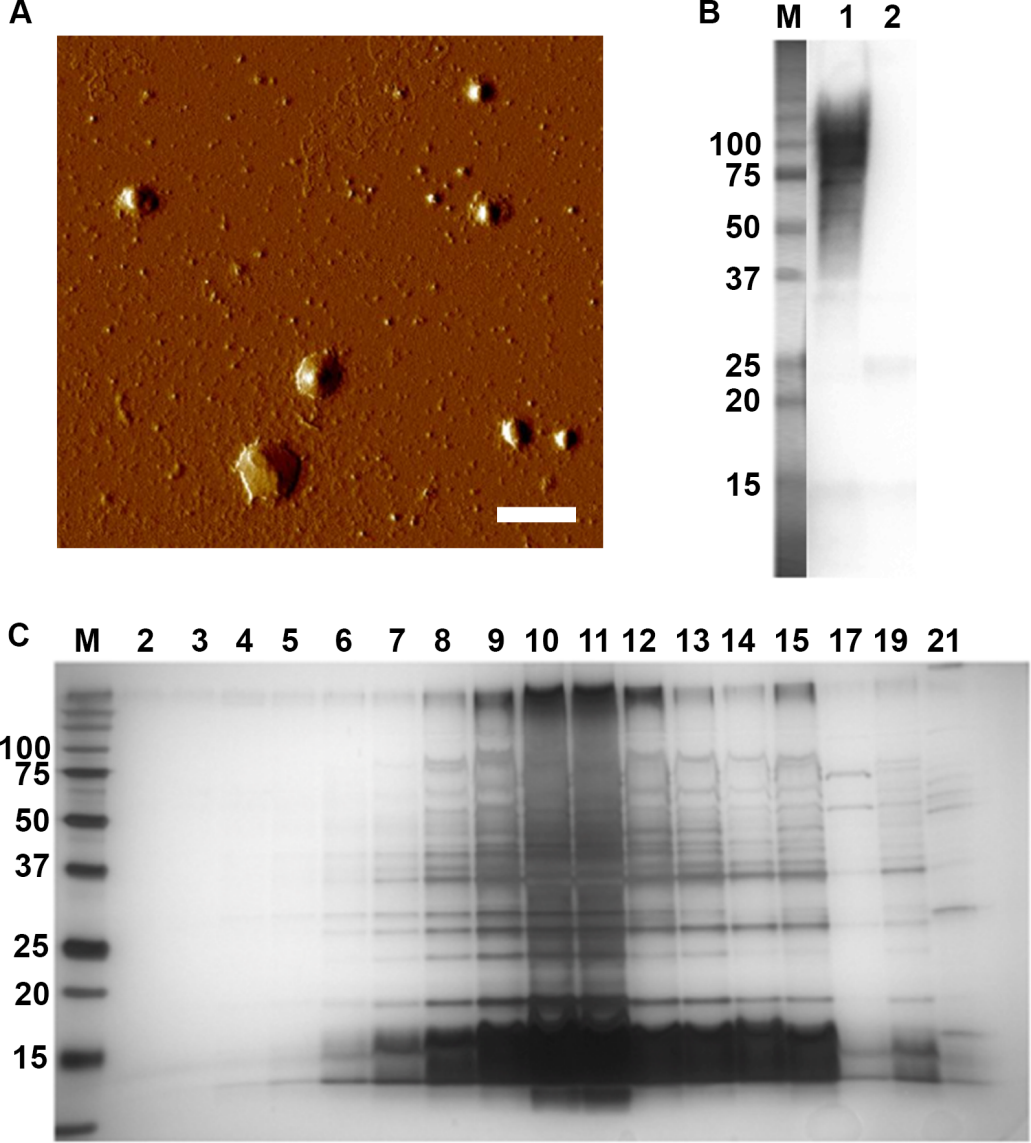
Purification of OMVs from *A*. *actinomycetemcomitans* strain 173. (A) Atomic force micrograph of OMVs obtained from strain 173 after density gradient centrifugation. Bar = 300 nm. (B) Reactivity of the isolated strain 173 OMVs (lane 1) to a rabbit antiserum made against whole *A*. *actinomycetemcomitans* serotype e bacterial cells. OMVs from strain D7SS (serotype a) are loaded in lane 2. Samples equal to 10 μg protein were applied on the gel. (C) SDS-PAGE analysis of density gradient fractions of strain 173 OMVs. Fractions (15 μl loaded on the gel) are numbered according to increasing density. The central fractions (8–12) were pooled and subject to LC-MS/MS analysis. The sizes (kDa) of the proteins in the pre-stained molecular weight marker (M) are indicated along the left side of the gels in panel B, and C. The images show one representative experiment.

To study outer membrane vesicle release by *A*. *actinomycetemcomitans*, vesicles were purified from strain 173 cells grown on agar (Fig 1A). The seroreactivity of the isolated OMVs was confirmed using a rabbit antiserum made against whole *A*. *actinomycetemcomitans* serotype e bacterial cells (Fig 1B). SDS-PAGE analysis of density gradient fractions of OMVs revealed a major population of OMVs, peaking approximately in the fractions 8 to 12 (Fig 1C), which is consistent with our earlier analyses of OMVs obtained from various *A*. *actinomycetemcomitans* strains [36]. To identify the proteins of the purified OMVs of strain 173, the central fractions (8–12) were pooled and used for further analysis by LC-MS/MS. The analysis of the OMV proteome included four independent preparations.

### Identification of *A*. *actinomycetemcomitans* strain 173 OMV proteins by LC-MS/MS, and their predicted subcellular distribution

**Fig 2:**
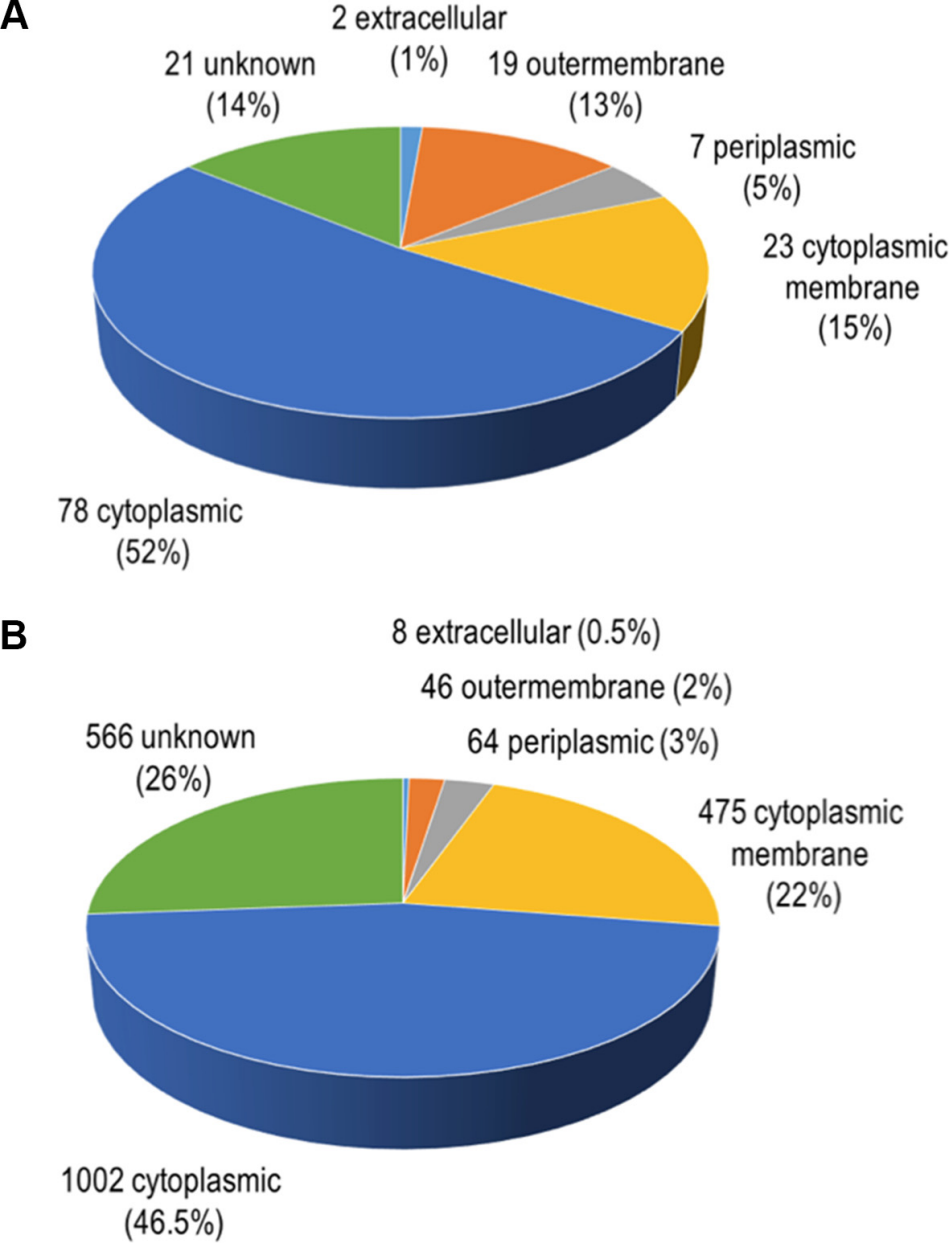
Proportions of predicted subcellular locations of the *A*. *actinomycetemcomitans* strain 173 OMV proteins identified by LC-MS/MS using PSORT3b. (A) Predicted subcellular locations of the 151 OMV proteins that were identified in at least three out of four independent vesicle preparations that were analyzed. (B) Predicted subcellular locations of all 2161 proteins encoded in the genome database of *A*. *actinomycetemcomitans* serotype e strain SC1083 that was used for the database searches of the LC-MS/MS analyses.

In total, 504 proteins were identified by LC-MS/MS in at least one of the four analyzed independent preparations of *A*. *actinomycetemcomitans* strain 173 OMVs. Out of these proteins, 151 were present in three of the four preparations analyzed, and defined as the OMV proteome in the present study (S1 Fig, S1 and S2 Tables). The number of 151 identified proteins is within the range of proteins detected in several high-throughput analyses of bacterial vesicle proteomes [28, 49]. To predict the subcellular localizations of the OMV proteins, PSORT3b 3.0 was used. According to our findings (Fig 2A), of the 151 proteins of the OMV proteome, two were predicted to be extracellular, 19 to be located in the outer membrane, 7 to be periplasmic, 23 to be located in the inner membrane, and 78 to be cytoplasmic. The relative proportions of proteins with predicted locations in the outer membrane and in the periplasm were found to be substantially higher among the 151 proteins than among the entire set of 2161 proteins encoded in the *A*. *actinomycetemcomitans* serotype e genome that was used for the database searches of the LC-MS/MS analyses (Fig 2B). This is in accordance with an enrichment of proteins derived from these subcellular fractions in OMVs. Identification of relatively large numbers of cytoplasmic proteins in OMVs was also observed in several other recent studies, even though the vesicles were purified using a density gradient [68–71]. Based on such observations it has been suggested that some cytoplasmic proteins may in fact be sorted into bacterial membrane vesicles [49, 72]. It is an area for future research to disclose mechanisms how and why these proteins might be selected for export via vesicles, and how vesicles incorporate other cytosolic material such as nucleic acid fragments [73–75]. One plausible mechanism might be the formation of double bilayered outer-inner membrane vesicles, as was demonstrated for *Shewanella vesiculosa* [76]. The observation that cytoplasmic proteins are present in OMV proteomes may also be due to that some of these proteins exhibit moonlighting capacities [77]. Moonlighting proteins comprise a subclass of multifunctional proteins in which more than one biochemical or biophysical function is contained within one polypeptide chain [78]. Moreover, presence of cytoplasmic proteins in OMV proteomes may be a result of cell lysis. However, among the proteins identified by LC-MS/MS (S1 and S2 Tables) we did not detect the cyclic nucleotide-binding domain protein (also known as cyclic AMP receptor protein [CRP]; GI:347994078), which is frequently used as a lysis marker in our studies for assessing the release of proteins by *A*. *actinomycetemcomitans* strains [18, 39, 79]. Notably, CRP was not detected in the extracellular medium of *A*. *actinomycetemcomitans* strain D7S cultivated as biofilm for up to 3 days, but could be released upon deliberate lysis of the bacterial cells [39, 79]. Therefore, absence of CRP in our present OMV preparations would argue against protein release due to bacterial lysis.

### Functional classification of proteins identified in *A*. *actinomycetemcomitans* strain 173 OMVs

**Fig 3:**
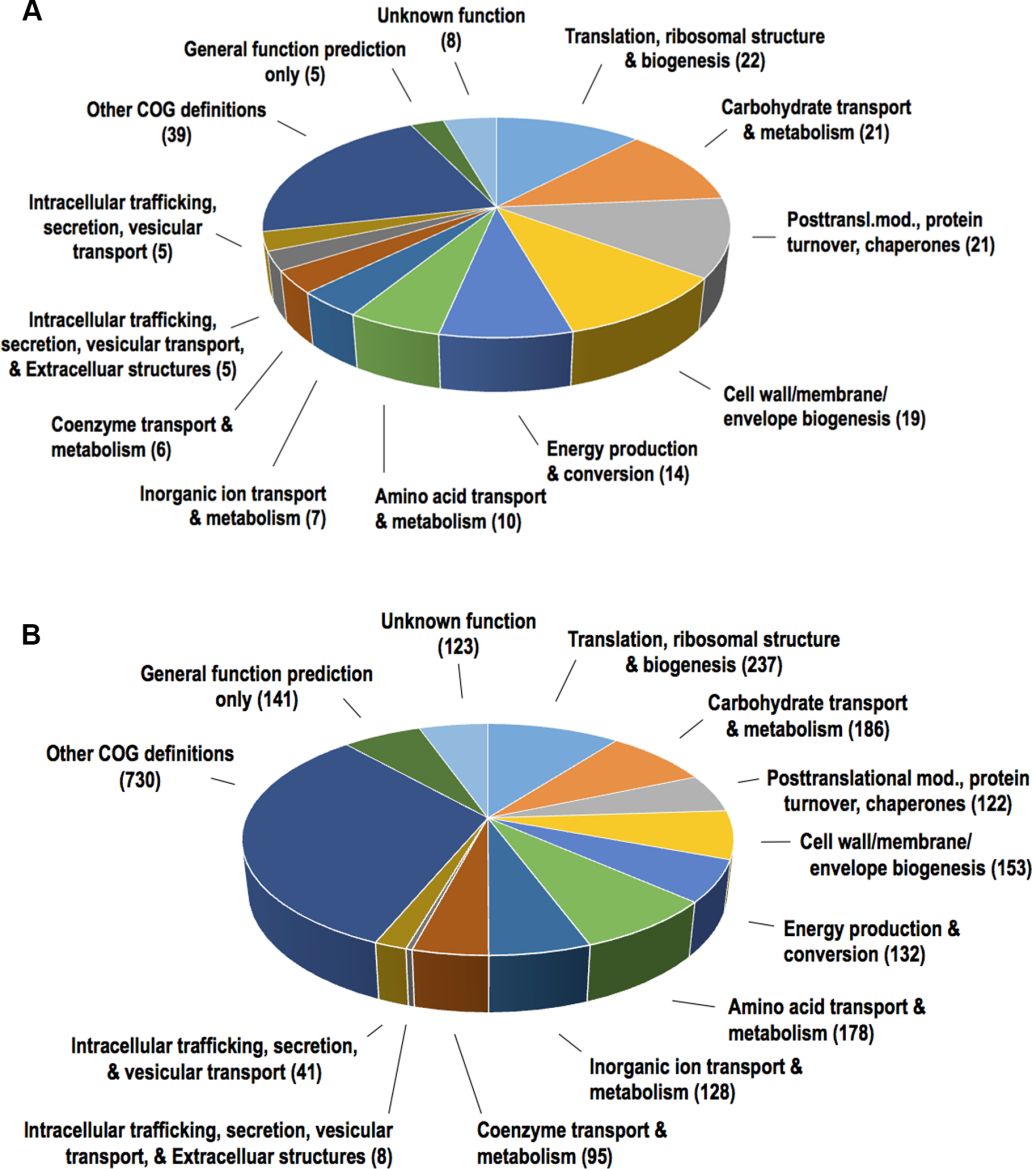
Functional classification of *A*. *actinomycetemcomitans* strain 173 OMV proteins. The 151 OMV proteins that were identified by LC-MS/MS in three out of four of the vesicle preparations were sorted according their COG groups as indicated. (A) This figure shows the proportions of the major groups of COG domains that were found in the OMV proteins, which were identified in this study. In total 182 COG domains were found in the 151 proteins. (B) This figure shows the corresponding COG domains that are present in the entire set of gene models of the genome of *A*. *actinomycetemcomitans* serotype e strain SC1083. The 2161 gene models of this strain contained 2274 COG domains.

To determine putative functions of the 151 proteins in the strain 173 OMV proteome, they were sorted according to their Cluster of Orthologous Groups (COG) definitions (Fig 3A). The group of 151 vesicle proteins had 182 of the 2274 COG definitions present in the database of the gene models of *A*. *actinomycetemcomitans* serotype e strain SC1083 (Fig 3B, S4 Table). The four largest COG groups recognized in the OMV proteome were found to be translation, ribosomal structure and biogenesis (22 of 237 COG definitions), carbohydrate transport and metabolism (21 of 186), post-translational modification, protein turnover, and chaperone activity (21 of 153), and cell wall, membrane, and envelope biogenesis (19 of 153). The relative abundance ratios of these COG groups were clearly higher in the OMV proteome than in the entire genome of *A*. *actinomycetemcomitans* serotype e strain SC1083. In particular, the group of post-translational modification, protein turnover, and chaperones stood out, accounting for 11.5% of the COG definitions in the experimental OMV proteome as compared to 5.5% of the entire genome. Taken together, these findings indicate that the OMV proteome may exhibit specific functions. This result is similar to COG categorization of the OMV proteomic content in a number of other species, e.g. *C*. *jejuni*, and *Edwardsiella tarda* [80, 81], and of cell envelope fractions of *A*. *actinomycetemcomitans* cells [82].

### Virulence-related mechanisms of *A*. *actinomycetemcomitans* vesicles as suggested by proteins identified in the strain 173 OMV proteome

To gain insight into the virulence potential of the strain 173 OMV proteome, and to obtain an overview of putative functional roles of the vesicles, the 151 proteins of the proteome identified by LC-MS/MS were manually searched for their earlier reported involvement in *A*. *actinomycetemcomitans* offense and defense, and in other bacteria. As summarized below, this screening revealed 49 proteins of particular interest (Table 1). As a complement to the literature search, the 151 vesicle proteins were also subject to *in silico* analysis using VirulentPred, an algorithm developed to predict putative novel virulence factors (S3 Table). Using this approach 60 of the OMV proteins (≈40%) were predicted to be associated with virulence, including most of the proteins revealed in the initial literature search. Our findings suggest that *A*. *actinomycetemcomitans* OMVs may exhibit several offensive and defensive functions analogous to those described in recent reviews, including immune evasion, drug targeting, and iron/nutrient acquisition [28–30].

**Table 1:** Virulence-related mechanisms of *A*. *actinomycetemcomitans* vesicles as suggested by proteins identified in the strain 173 OMV proteome. The 151 proteins of the strain 173 OMV proteome identified by LC-MS/MS were manually searched for their earlier reported involvement in *A*. *actinomycetemcomitans* offense and defense, and in other bacteria. The listed proteins are discussed in the main text.

Type of OMV function	Shown for *A*. *actinomycetemcomitans* protein^a)^	Shown for paralogue in other bacteria, or predicted based on protein amino acid sequence
Immunoreactivity and/or proinflammatory activity	GroEL, LtxA, OmpA-like protein, Omp18/16, Omp39, Omp100, Pal, RcpA, RcpC, TadA, TadD, TadZ, YaeT	OmpA
Cytotoxicity	LtxA, GroEL	OmpA-like protein, OmpA
Adhesion/invasion	Omp100, RcpA, RcpB, RcpC, TadA, TadD, TadE, TadF, TadG, TadZ	
Immune evasion	BilR1, Omp100	Factor H-binding protein, OmpA-like protein, OmpA
Drug targeting	TdeA	AcrA, DNA gyrase, elongation factor G, organic solvent tolerance protein, penicillin-binding protein 1A, ribosomal proteins (n = 16), RNA polymerase, TolB
Scavenging of iron and nutrients		Ferric transporter ATP-binding subunit, Ferritin-like protein, Omp64, putrescine/spermidine ABC transporter ATPase protein, TonB-dependent siderophore receptor

^a)^ Full protein descriptions as appearing in the genome database used, and their accession numbers are listed in S1, S2, and S3 Tables.

### Immunoreactivity and proinflammatory activity

**Fig 4:**
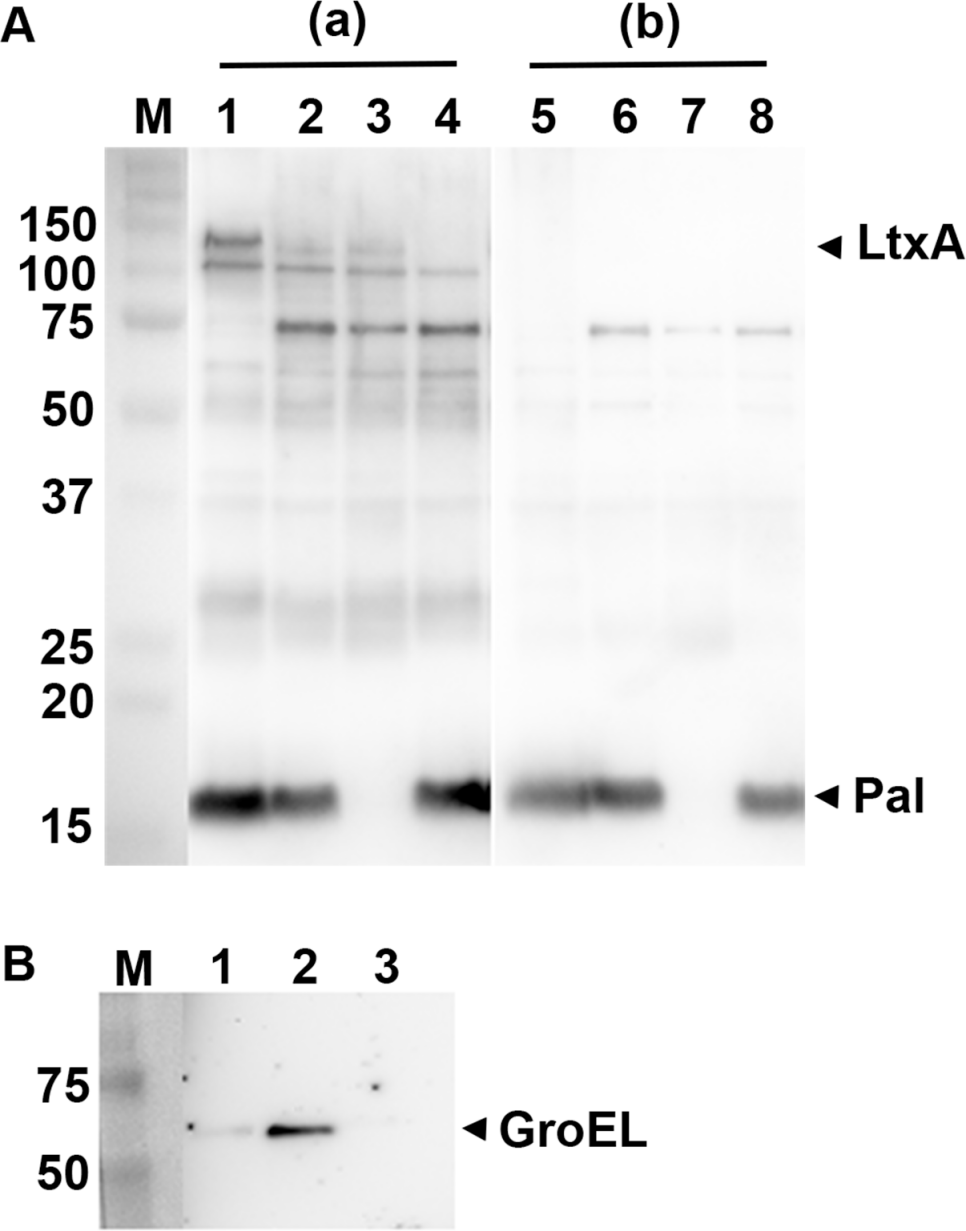
Immunoreactivity of *A*. *actinomycetemcomitans* OMVs. (A) Western blot analysis of reactivity of human sera with OMVs obtained from the *A*. *actinomycetemcomitans* strains 173 (lanes 1 and 5), D7SS (lanes 2 and 6), D7SS-p (Δ*pal*; lanes 3 and 7), and D7SS Δ*ltxA* Δ*cdtABC* (lanes 4 and 8). An *A*. *actinomycetemcomitans*-responsive serum from a periodontitis subject (a) and from a periodontally healthy, *A*. *actinomycetemcomitans*-negative individual (b) was used for the immunodetection, respectively. (B) Western blot detection using a polyclonal antiserum specific for *E*. *coli* GroEL. Vesicles obtained from *A*. *actinomycetemcomitans* strains 173 (lane 1), and D7SS (lane 2), and from *S*. *aureus* strain 8325–4 [91] (lane 3) were analyzed. Samples equal to 10 μg protein were applied on the gels. Reactive bands corresponding to LtxA, Pal, and GroEL are indicated with arrowheads. The sizes (kDa) of the proteins in the pre-stained molecular weight marker (M) are indicated along the left side.

According to our data, the strain 173 OMV proteome contains several proteins earlier demonstrated to act as immunoreactive antigens in the human host. LtxA, Omp39, RcpA (referred to as GspD in the database used; GI:347992426), RcpC (FlpC; GI:347992425), TadA, TadD, TadZ (FlpD; GI:347992428), and YaeT (also known as BamA) were recognized exclusively by antibodies in sera from subjects with a proven *A*. *actinomycetemcomitans* infection [47]. Moreover, the OmpA-like protein (GI:347991272; also referred to as Omp29 or Omp34), which is a major protein component of *A*. *actinomycetemcomitans* OMVs [40], is immunoreactive in patients with periodontitis, and so are Omp18/16, Omp100 (GI:347992912; also referred to ApiA) [53, 83–85]. The ability of Pal to elicit proinflammatory responses in human cells has been demonstrated [39, 79]. There is also evidence that Omp100 induces the expression of proinflammatory cytokines *in vitro* [86]. Finally, GroEL is implicated as an immunodominant *A*. *actinomycetemcomitans* antigen, which promotes epithelial cell proliferation [38, 87–89]. Carriage of GroEL by OMVs also suggests the possibility that vesicles may contribute to alveolar bone resorption [87, 90]. Western blot analysis was used to corroborate our LC-MS/MS findings. In contrast to a control serum from an *A*. *actinomycetemcomitans*-negative subject, which mainly produced faint signals, an *A*. *actinomycetemcomitans*-responsive patient serum strongly reacted to a number of proteins in strain 173 OMV preparations, including LtxA and Pal (Fig 4A). The observation that Pal is a major immunoreactive antigen of the vesicles is in line with patterns of patient serum reactivity to *A*. *actinomycetemcomitans* outer membrane protein preparations [53]. Likewise, the relatively strong reactivity of the control serum to Pal (Fig 4A) is consistent with presence of cross-reacting antibodies to Pal produced by other oral and/or non-oral Gram-negative species [53]. We deduced that the ≈60 kDa immunoreactive band (Fig 4A) might correspond to GroEL, as this protein was detected in the OMVs using a GroEL-specific rabbit antiserum (Fig 4B). A membrane vesicle preparation from *Staphylococcus aureus* strain 8325–4 [91] was here loaded as a negative control for GroEL, as this protein, which appears to be essential for bacterial growth [92], was not identified in *S*. *aureus* membrane vesicle proteomes [93, 94]. In summary, our observations support the notion that the *A*. *actinomycetemcomitans* OMV proteome represents a potent source of multiple proinflammatory stimulants. Our data also suggest a potential role of *A*. *actinomycetemcomitans* OMVs as immunogens for vaccines against periodontal disease, in a similar manner as vesicles from *Porphyromonas gingivalis*, which carry several proteins exhibiting strong immunogenicity in a mouse vaccine model [95, 96].

### Cytotoxicity: Leukotoxic activity

**Fig 5:**
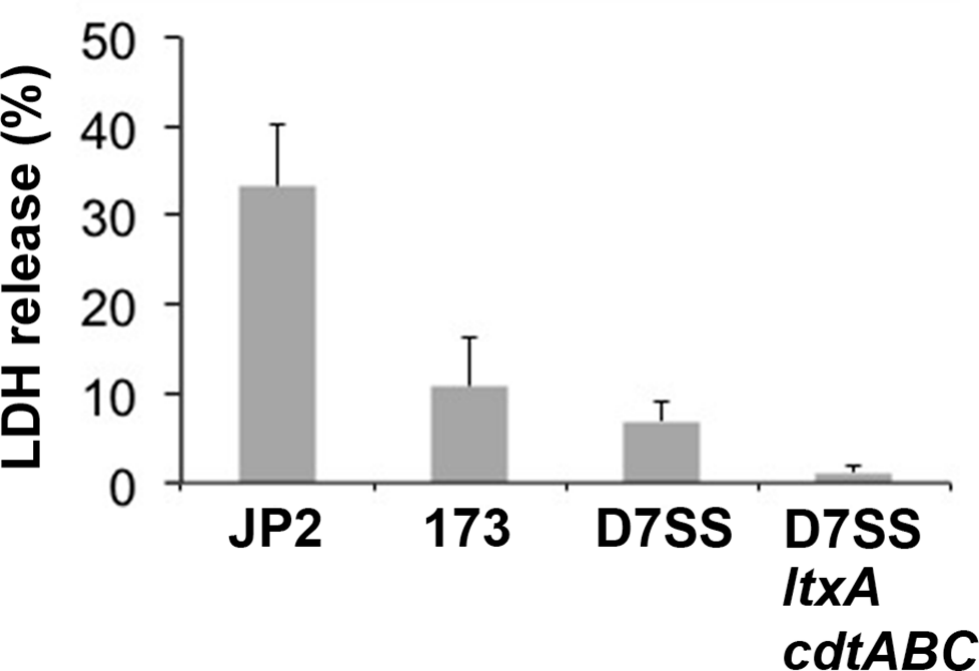
Leukotoxic activity of OMVs obtained from the indicated *A*. *actinomycetemcomitans* strains. THP-1 cells were incubated with OMVs (final concentration: 100 μg/ml) for 120 min. Release of LDH was determined as described in Materials and Methods, and is expressed as % of the maximal release (100%) caused by incubation with 0.1% Triton X-100. The results shown are means + standard error of the means (SEM) from three independent experiments.

LtxA was identified in OMVs from the serotype e strain 173, which is consistent with earlier reports assessing vesicles derived from serotype a, and b strains [36, 40]. To confirm the presence of biologically active LtxA in the strain 173 OMVs, lysis of THP-1 cells incubated with vesicles was quantitated using an LDH release assay (Materials and Methods). This revealed that OMVs from strain 173 exhibited leukotoxic activity, albeit at a level approximately four times lower than vesicles from the highly leukotoxic strain JP2 (Fig 5). This relative difference in leukotoxicity is consistent with observations assessing extracts from these two *A*. *actinomycetemcomitans* strains [17]. The leukotoxicity of strain 173 OMVs was similar to that of vesicles from D7SS, which is considered a minimally leukotoxic strain [97]. Among the identified vesicle proteins, also GroEL may contribute to OMV cytotoxicity as judged by the deleterious effect of this protein on human epithelial cells [38]. It is not known if the OmpA-like protein Omp29/Omp34, or its paralogue OmpA (GI:347992918) may play a role in OMV cytotoxicity towards human cells as has been demonstrated with the nosocomial pathogen *Acinetobacter baumanii* [98]. We note that CDT was not identified in the strain 173 OMV proteome, whereas this toxin is evidently released in association with vesicles by a number of other *A*. *actinomycetemcomitans* strains of serotypes a, b, and c [36]. This is consistent with observations that strain 173 lacks cytolethal distending activity, which appears to be due to an incomplete subset of the *cdt*-encoding genes [50].

### Adhesion and invasion

A number of proteins that may contribute to adherence of vesicles to host cells were identified in the strain 173 OMV proteome. Omp100 promotes adhesion of *A*. *actinomycetemcomitans* cells, and their invasion of human gingival keratinocytes [86, 99]. Moreover, RcpA, RcpB, RcpC, RadZ, TadA, TadD, TadE, TadF, and TadG are components of the *A*. *actinomycetemcomitans tad* (tight adherence) gene locus, which mediates adhesion, and is required for virulence in a rat model for periodontal disease [100]. Recent evidence has supported the internalization of *A*. *actinomycetemcomitans* OMVs into non-phagocytic human cells such as gingival fibroblasts [41]. However, it is not known whether specific OMV-associated adhesins would be involved in adherence of the vesicles to host cells, comparable to the roles of *Helicobacter pylori* BabA, SabA, and VacA, *M*. *catarrhalis* UspA1, and *Pseudomonas aeruginosa* aminopeptidase [28, 29].

### Immune evasion

A subset of proteins that may play a role in immune suppression and evasion were identified in the strain 173 OMV proteome. A hypothetical protein (GI: 347993884) exhibits ≈86% amino acid identity to BilR1, which is a recently recognized *A*. *actinomycetemcomitans* IL-1β-binding lipoprotein [101]. Carriage of such receptor by OMVs suggests that they might enhance bacterial immune evasion by sequestering IL-1β to reduce inflammation. *A*. *actinomycetemcomitans* strains are typically resistant to killing by human serum [86], and our present results suggest the possibility that OMVs may play a role in serum survival. For example, Omp100 was demonstrated to be important for serum resistance of *A*. *actinomycetemcomitans* strain IDH781 (serotype d), which appeared to be a result of Omp100-mediated binding of Factor H to the bacterial surface [86]. Moreover, we identified factor H-binding protein, a paralogue to the protein of *Neisseria* spp. that is critical for survival of meningococci in the human host [102]. Whether the *A*. *actinomycetemcomitans* factor-H binding protein might play a similar functional role is not known. Also OmpA might interact with human serum factors to contribute to serum resistance, analogously to findings obtained using *A*. *baumannii*, and *E*. *coli* bacterial cells [103, 104].

### Drug targeting

The strain 173 OMV proteome was found to contain proteins that could bind to/serve as targets for drugs. There are antibiotics that directly target DNA gyrase [105], elongation factor G [106], organic solvent resistance protein (also known as LptD or Imp/OstA) [107], penicillin-binding protein 1A [108], and RNA polymerase [109]. Also, TolB is a potential drug target [110]. Identification of ribosomal proteins in the OMV proteome is in accordance with detection of rRNA fragments in OMVs from other organisms [74]. Our findings are also consistent with other proteomics studies on vesicles from various bacterial species, e.g. *S*. *aureus* and *P*. *aeruginosa* [93, 94, 111]. For example ribosomal protein S12 was identified in such studies, which is bound by several antibiotics including aminoglycosides [111, 112]. Whether presence of such drug target proteins in vesicles would play a functional role during infection is not known. However, intriguingly, it was shown that the aminoglycoside gentamicin bound to the surface of tentative OMVs of *P*. *aeruginosa* [113]. Hence, vesicles may have the ability to bind directly with some antibiotics, thereby lowering their local free concentrations, contributing to protection of the bacterial cell. Efflux systems, analogously might function as binding proteins for their substrates in OMVs. Among such proteins in the OMV proteome, TdeA is a TolC-like protein that is required for secretion of LtxA, and resistance to antimicrobial compounds. As judged by homology, this protein may represent a component of a drug efflux porin system in *A*. *actinomycetemcomitans* [114]. Similarly, AcrA is a component of the tripartite AcrAB-TolC multidrug efflux pump, which is well characterized in *E*. *coli* [115, 116].

### Scavenging of iron and nutrients

Ferritin-like protein, ferric transporter ATP-binding subunit, putrescine/spermidine ABC transporter ATPase protein, a TonB-dependent siderophore receptor, and Omp64, which has a TonB-like iron-binding site in the C-terminus [83] were also identified in the strain 173 OMV proteome. This suggests that the vesicles could play a role in scavenging iron and nutrients, which might be subsequently released in the vicinity of and provided to bacterial cells. This is consistent with a number of previous proteomics studies of OMVs, identifying vesicle-associated TonB-dependent receptors, metal ion binding proteins, ABC transporters, and machineries for ATP synthesis, supporting the idea that OMVs may collect and concentrate scarce ions and nutrients for the consumption of bacterial cells [28, 49, 71, 117, 118]. Interestingly, such a role of OMVs, also allowing intraspecies nutrient transfer was recently demonstrated regarding carbon flux between several species of bacteria and cyanobacteria [119].

## Conclusions

In conclusion, we have characterized the OMV-associated proteome of the rough *A*. *actinomycetemcomitans* serotype e strain, 173 by LC-MS/MS, and used manual literature search and the VirulentPred algorithm to assess the virulence potential of this subproteome. To the best of our knowledge, this work represents the first proteomic study of purified *A*. *actinomycetemcomitans* OMVs. Apparently, the virulence potential may be subject to variation among *A*. *actinomycetemcomitans* strains, including their released OMVs. As an example, CDT was not identified in the strain 173 OMV proteome, whereas this toxin is evidently released in association with vesicles by a number of other *A*. *actinomycetemcomitans* strains. Nevertheless, our results suggest that a reasonably large part of the OMV proteins may contribute to the virulence potential of the vesicles. Such proteins include established virulence factors like LtxA, and major antigens such as Pal, which is also suggesting a potential role of *A*. *actinomycetemcomitans* OMVs as immunogens for future vaccines against periodontal disease. Moreover, by identifying numerous additional putative virulence-related proteins in the vesicle proteome, our work lays the molecular groundwork for novel mechanistical studies that for instance could elucidate the roles of *A*. *actinomycetemcomitans* OMVs in immune evasion, drug targeting, and iron/nutrient acquisition. Finally, as compared to several recent high-throughput proteomics studies on OMVs, we identified relatively large numbers of cytoplasmic proteins in the vesicles, and we conclude that it is an area of interest for future research to disclose mechanisms how such proteins might be targeted for vesicle export.

## Supporting Information

S1 Fig Venn diagram obtained from the comparison of the LC-MS/MS-identified proteins of the four independent *A*. *actinomycetemcomitans* strain 173 OMV preparations. In total 504 proteins were identified, out of which 151 were present in at least three out of the four preparations that were analyzed.
(TIF)

S1 Table Proteins identified by LC-MS/MS in at least one of the four independent OMV preparations of *A*. *actinomycetemcomitans* strain 173. In total, 504 proteins were identified.
(XLSX)

S2 Table Proteins identified by LC-MS/MS in at least three out of four independent OMV preparations of *A*. *actinomycetemcomitans* strain 173. A total of 151 proteins were identified.
(XLSX)

S3 Table VirulentPred screening of the 151 proteins in the *A*. *actinomycetemcomitans* strain 173 OMV proteome. Where available, references are included that support virulence-related activities of the *A*. *actinomycetemcomitans* proteins and/or their paralogues in other bacterial species.
(XLSX)

S4 Table COG definitions for all gene models of *A*. *actinomycetemcomitans* serotype e strain SC1083 according to NCBI. (XLSX)
