# Supplementary figures and images for: Proteomics of Aggregatibacter actinomycetemcomitans Outer Membrane Vesicles
Source: PLoS One. 2015 Sep 18;10(9):e0138591. doi: 10.1371/journal.pone.0138591 (PMC4575117; doi:10.1371/journal.pone.0138591)

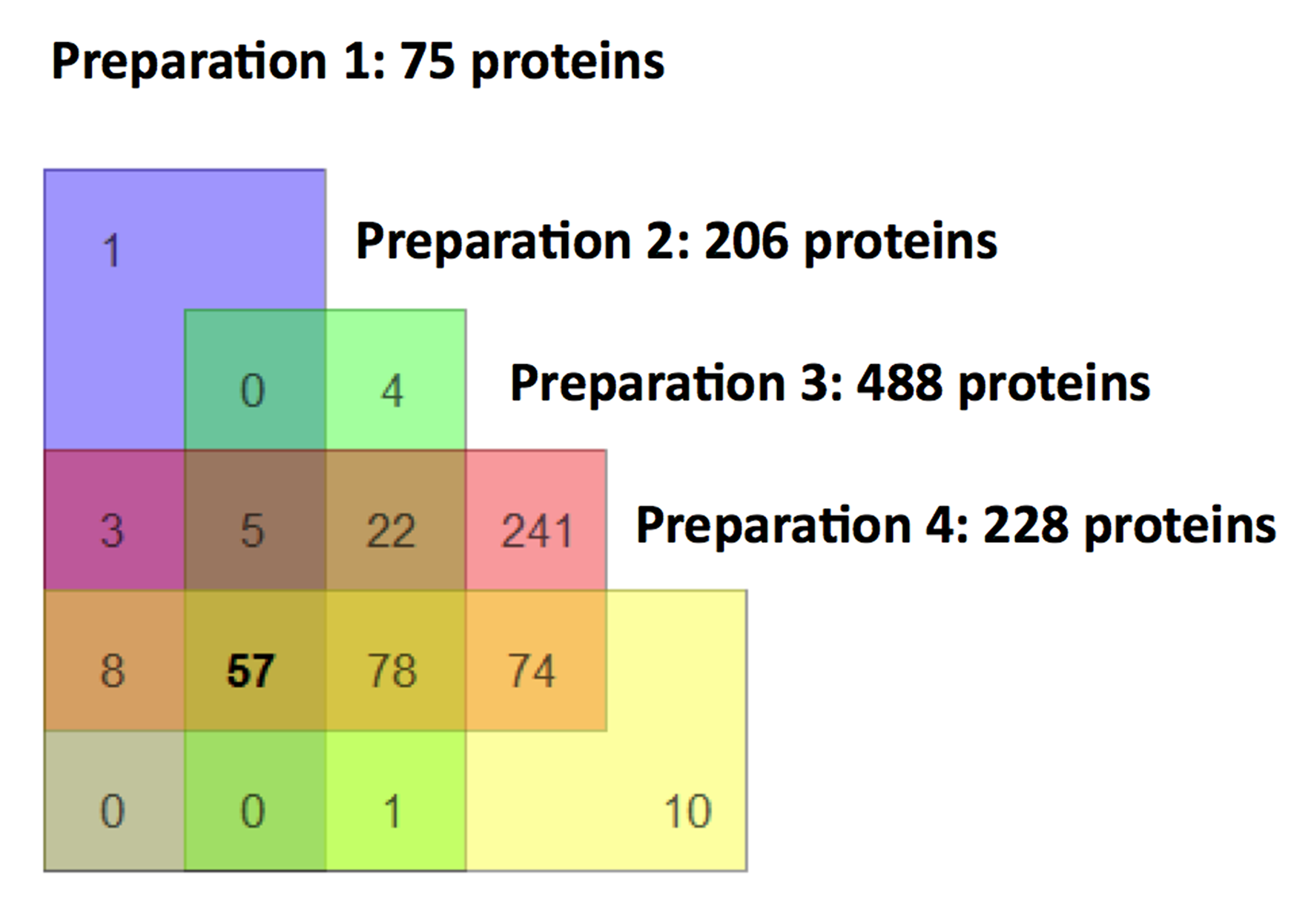

Supplement: S1 Fig — In total 504 proteins were identified, out of which 151 were present in at least three out of the four preparations that were analyzed. (TIF) [file pone.0138591.s001.tif]
